# Detection, Detrimental Effects, and Transmission Pathways of the Pathogenic Bacterium Acaricomes phytoseiuli in Commercial Predatory Mites

**DOI:** 10.1128/spectrum.02654-22

**Published:** 2022-11-02

**Authors:** Zhongqiu Xie, Ary A. Hoffmann, Bo Zhang, Xuenong Xu

**Affiliations:** a Institute of Plant Protection, Chinese Academy of Agricultural Sciences, Beijing, China; b Bio21 Institute, School of Biosciences, University of Melbourne, Parkville, Victoria, Australia; c Key Laboratory of Natural Enemies Insects, Ministry of Agriculture and Rural Affairs, Beijing, China; South China Agricultural University

**Keywords:** biological control, vertical transmission, cross-species infection, fluorescence localization, *Neoseiulus californicus*

## Abstract

Arthropod pathogens and other microorganisms have been documented from mass production systems aimed at producing natural enemies for pest control. If losses due to pathogens are encountered in such systems, they could lead to uneconomical production of natural enemies and/or a loss of predator quality for effective field control of target pests. Here, we identify and describe the laboratory transmission of a bacterial pathogen, Acaricomes phytoseiuli, in a Chinese strain of the local predatory mite Neoseiulus californicus following contact with Phytoseiulus persimilis, a predatory mite imported for the control of small sap-sucking pests in greenhouses. Analysis of the 16S rRNA gene and whole-genome sequences of *A. phytoseiuli* isolated from the Chinese strain of *N. californicus* showed 99.6 and 99.78% similarity, respectively, to the pathogen isolated from a European population (DSM14247 strain). This is the first report of *P. persimilis* infected with *A. phytoseiuli* outside Europe and transmitting to a local predatory mite species. *A. phytoseiuli* severely damaged local *N. californicus,* leading to a dorso-ventrally flattened body and reduced prey consumption and reproduction as well as early death. Through fluorescence *in situ* hybridization, *A. phytoseiuli* was shown to accumulate in the digestive tract of mites and in the oviductal bulb of adult females. Infected males had no obvious symptoms, but they still were able to pass on the infection to healthy females through contact and mating. The pathogen was transmitted vertically to offspring by either infected parent through adherence to eggshells. *A. phytoseiuli* could also persist in other herbivorous arthropods from the same habitat, suggesting wider potential risks. Our study highlights pathogen risk to predators in local biocontrol industries through pathogen spread from imported material.

**IMPORTANCE** Predatory mites are important natural enemies for biological control of pests, but mass rearing of the mites can be affected by pathogens. In particular, the mite pathogen Acaricomes phytoseiuli may pose a threat to predatory mite production, and we have now detected this pathogen in China. We explored the pathogen’s transmission within species, its ability to transfer to a locally important predatory mite species, and symptoms following transfer. The detection of *A. phytoseiuli* and its ability to transfer to a local predator where it reduces performance highlight the importance of ongoing monitoring and hygiene in the predatory mite industry.

## INTRODUCTION

Arthropods and microbes interact in various ways, with the microorganisms functioning as mutualists, pathogens, or commensals, depending on the nature of their interactions with the host (1). Environmental microbes can become opportunistic pathogens of their arthropod hosts, particularly when arthropod hosts develop under overcrowded conditions, leading to hosts encountering much higher doses of pathogens than they would likely encounter normally (2). This is an important consideration for natural enemies of arthropods, which are increasingly being intensively produced under crowded conditions for release to control pests in various agroecosystems (3).

Predatory mites represent important predators that have been widely used in biological control for more than half a century worldwide. In particular, Phytoseiulus persimilis Athias-Henriot has become one of the mainstays of integrated management of spider mites in greenhouses in Europe since the 1960s (3, 4). However, the effect of pathogens on predator mite quality is often ignored in mass rearing situations, even though some pathogens are known to affect the performance of their mite hosts. Acaricomes phytoseiuli is the first report bacterial pathogen in *P. persimilis* isolated from host populations in Europe (5), resulting in host mites insensitive to herbivore-induced plant volatiles, known as “nonresponding syndrome,” as well as ceasing prey consumption and oviposition until eventual death (6). *P. persimilis* infected by *A. phytoseiuli* therefore presents a risk in integrated pest control (IPM) programs based on release of mass-reared mites. Moreover, as commercially produced *P. persimilis* mites are traded worldwide, but other predatory mites can be important to industries locally, there is the potential for pathogens to spill over from imported species to local commercially important species where the microbes may have detrimental effects.

In China, *P. persimilis* was first introduced from Sweden as a commercial strain in 1975 (7). There have been subsequent introductions from Europe to local commercial producers and further exchanges among research institutions and between these and commercial producers in the past few decades (8, 9). Besides widespread release of *P. persimilis*, the suitability of mass rearing of many indigenous predatory mites has also been explored and other mite species are now produced for different crop zones targeting various pests in China (9): these include Neoseiulus californicus McGregor (10), a particularly efficient predator of spider mites (11), citrus red mites (12), and thrips (13) on vegetables and fruits. To improve control of a range of mite pests, *N. californicus* has been used in a complementary manner with *P. persimilis* in the field.

The *A. phytoseiuli* infection has so far only been documented in *P. persimilis* populations in Europe, but not in *P. persimilis* or other predatory mite species in the United States and Australia (5), even though predatory mites have been moved across continents for several decades. Here, we now report the first detection of *A. phytoseiuli* on *P. persimilis* populations reared over 10 years in our lab. The pathogen showed a high level of molecular similarity with European isolates. We then demonstrate the capability for the pathogen to transfer to *N. californicus*, where it had a substantial negative effect on female reproduction. We also characterize the mode of transmission of the pathogen within species and its potential transfer to other arthropods.

## RESULTS

### Acaricomes phytoseiuli detected in area of Asia for the first time.

**FIG 1:**
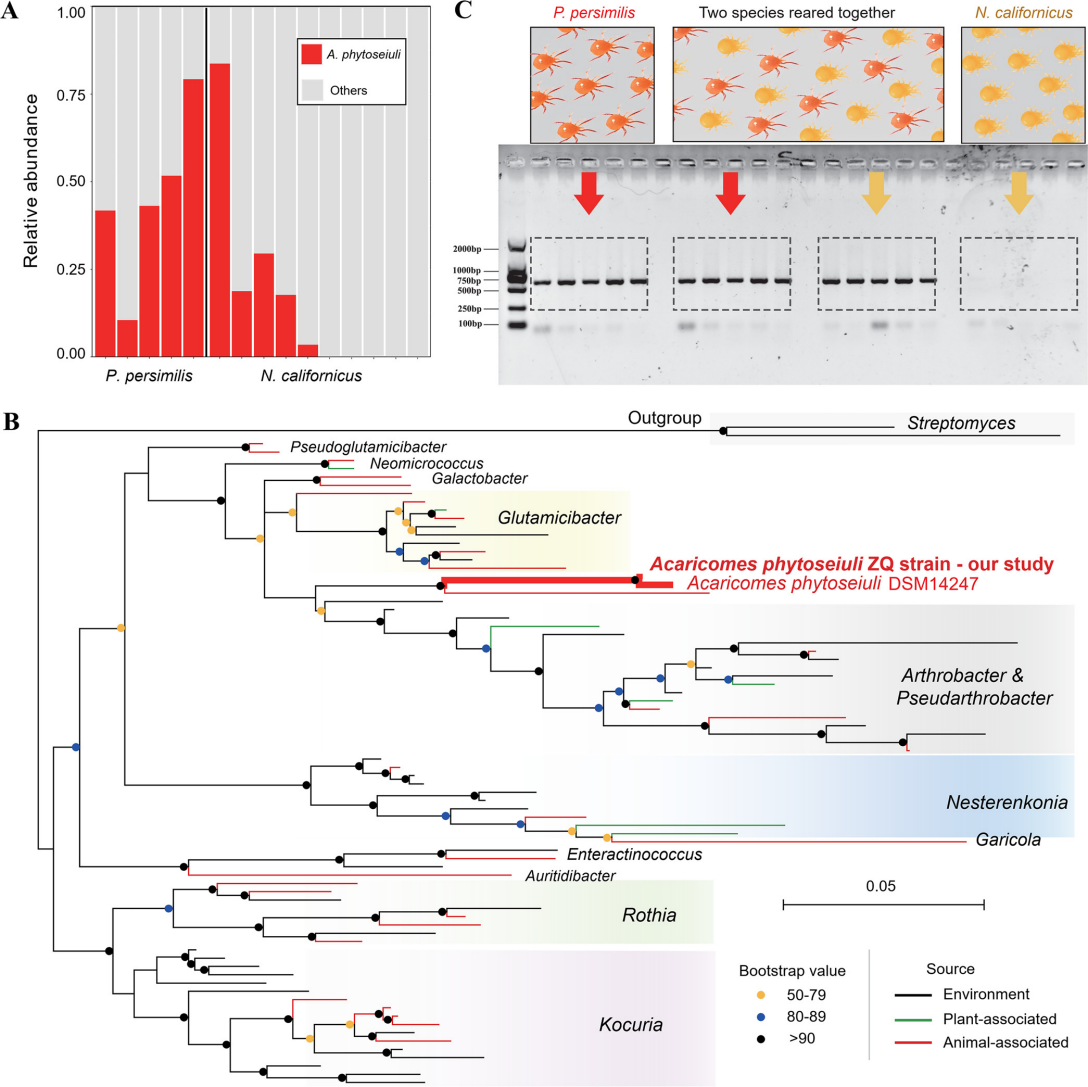
Detection of *A. phytoseiuli* in two species of predatory mites, *P. persimilis* and *N. californicus*. (A) Relative abundance of *A. phytoseiuli* from 5 and 10 lines of *P. persimilis* and *N. californicus*, respectively; (B) maximum likelihood tree of 16S rRNA sequences among *Micrococcaceae*. Bar colors indicate bacteria isolated from environments (black), plants (green), or animals (red). Relative bootstrap values are represented by circles at each node with colors. (C) Presence of *A. phytoseiuli* in the original lines and mixed rearing lines of two mite species, including (from left to right) *P. persimilis* cultured by itself, mixed cultures of *P. persimilis* and *N. californicus*, and *N. californicus* cultured by itself.

We selected 5 *P. persimilis* and 10 *N. californicus* laboratory lines for screening of the microbial community by 16S rRNA amplicon sequencing. Among the *N. californicus* lines, 5 shared an insectary with *P. persimilis*, while the other 5 were located in a separate insectary. *A. phytoseiuli* was identified in all *P. persimilis* lines and 5 *N. californicus* lines (Fig. 1A); all *A. phytoseiuli*-positive *N. californicus* lines were from the insectary shared with *P. persimilis*. The relative abundance of *A. phytoseiuli* in the microbial community as assessed from read number was high and constituted >75% of reads in two of the lines tested for each of the species (Fig. 1A).

The *A. phytoseiuli* strains from Chinese populations of *P. persimilis* and *N. californicus* showed 100% similarity of 16S rRNA sequence (MZ361977 and MZ363838), while they were 99.6% identical (1,326/1,331 bp) to the sequence of the DSM14247 strain isolated from a European population (NR_042334) (14). Phylogenetic analysis indicated that the isolates from *N. californicus* named as ZQ strains belonged to the *Acaricomes* genus, with the nearest phylogenetic neighbor being Haematomicrobium sanguinis (NR_044399), a pathogen isolated from human blood (Fig. 1B and see Fig. S1 in the supplemental material). The average nucleotide identity (ANI) analysis suggested that the ZQ strain was closely related to the DSM14247 strain (GCA_000376245.1) with 99.78% identity (PRJNA886762) (Table S1), possessing a 2.37-Mb genome with 2,194 genes. To investigate the potential virulence factors affecting host mites, we characterized 176 genes from the Virulence Factor database (VFDB) and 140 genes from Pathogen-Host Interactions database (PHI-base) by protein sequence BLAST searches. A total of 56 intersecting virulence genes were found, which involved intercellular toxins (proteases), secretion systems and effectors (type I secretion system [T1SS], T4SS, and T6SS), and response regulators and transporters (Table S2), suggesting potential molecular interactions between the pathogen and its hosts.

To clarify whether *A. phytoseiuli* was an original pathogen in local *N. californicus*, we amplified the 16S rRNA gene of this bacterium in four wild populations from various plant hosts (Tables S3 and S4). None of the samples in wild populations were found to harbor *A. phytoseiuli*. Then we intentionally set up containers to monitor whether *A. phytoseiuli* could horizontally transfer between laboratory species in a confined space. We found that all five replicates of an uninfected culture of *N. californicus* (Fig. 1C, indicated in yellow) became infected after 2 weeks of being held together in rearing boxes with *A. phytoseiuli-*positive *P. persimilis* (red) (Fig. 1C).

### Pathogenicity of *A. phytoseiuli* to *N. californicus*.

**FIG 2:**
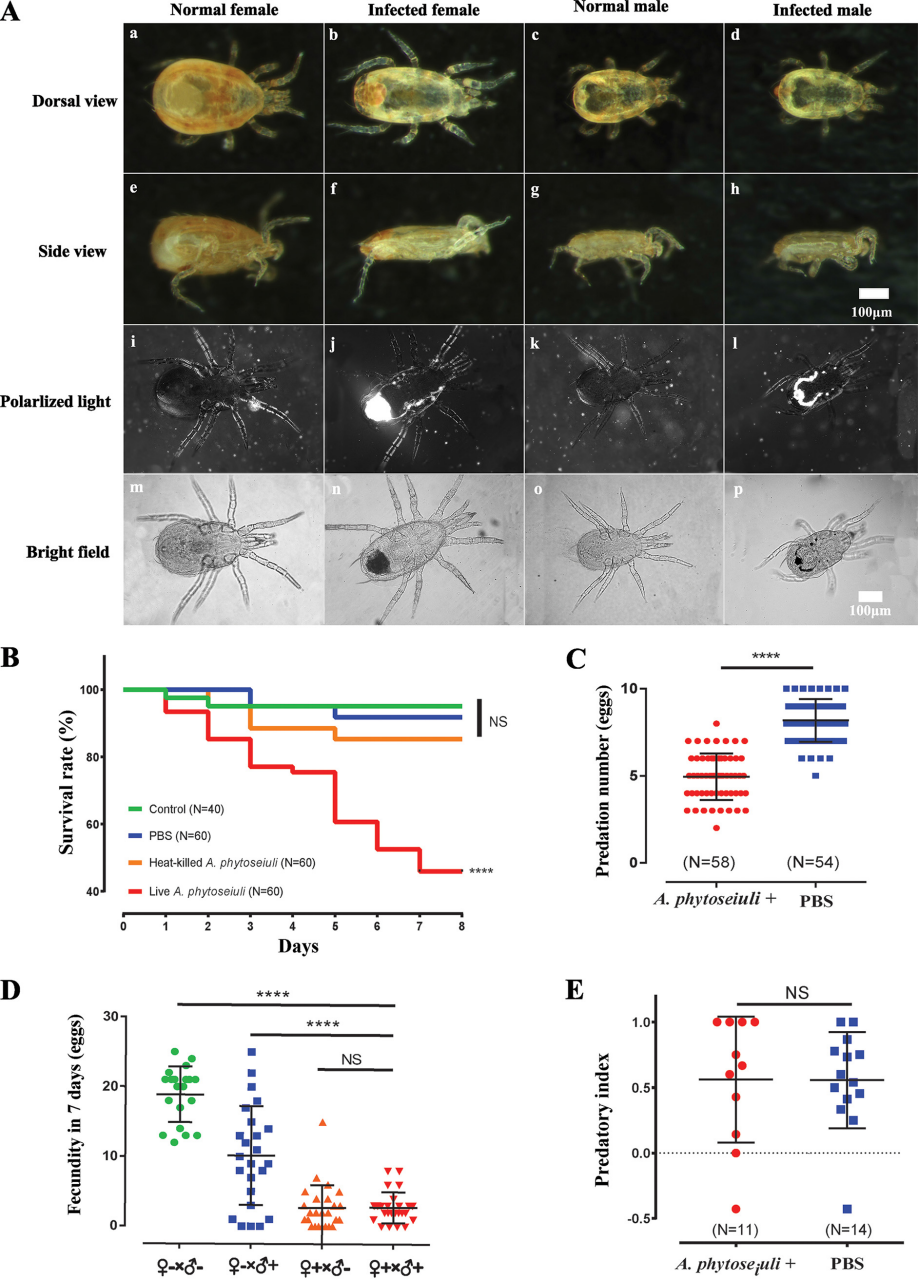
Symptoms of *N. californicus* infected by *A. phytoseiuli*. (A) Graphs of adult mites of both sexes with and without pathogen under stereoscopic microscope and light microscope. Bright color in panel j and l insets represents locations of exogenous microbial accumulation. (B) Survival rate of *N. californicus* after being treated with a 1× PBS suspension of live- and heat-killed *A. phytoseiuli*; (C) *T. urticae* egg consumption by *N. californicus* infected with and without *A. phytoseiuli*; (D) first week fecundity of *N. californicus* mated in four combinations. “−” indicates clean adults, and “+” indicates adults infected by *A. phytoseiuli.* (E) Predatory index of *N. californicus* infected with and without *A. phytoseiuli*. Each group/dot indicates the preference of 20 female mites. ****, *P* < 0.0001; NS, not significant.

Using *in vivo* virulence assays, we found that *A. phytoseiuli* was pathogenic to new host *N. californicus*, which was infected after exposure to a microbial suspension (Fig. 2). When infected with *A. phytoseiuli*, female adults were affected morphologically, becoming dorso-ventrally flattened, with a less intense body color and with discoloration in the opisthosoma compared to healthy individuals (Fig. 2A, panels b and f). In contrast, infected males were only lighter in color than uninfected males (Fig. 2A, panels d and h). We also found that both the infected male and female adults had dark stains in the opisthosoma when observed on glass slides (Fig. 2A, panels n and p). Under polarized light, bright areas were obvious to represent crystal precipitations in the rectum and Malpighian tubules in infected mites (Fig. 2A, panels j and l), in contrast to normal females and males (Fig. 2A, panels i and k).

*A. phytoseiuli* caused >50% mortality in *N. californicus* within a week (Fig. 2B) (Kaplan-Meier survival curve, *P* < 0.001), whereas exposure to heat-killed *A. phytoseiuli* did not affect the survival rate of mites, suggesting a detrimental effect of bacterial proliferation on their hosts. The *A. phytoseiuli* infection also significantly reduced the predatory capacity of *N. californicus* from 8.18 to 4.95 spider mite eggs consumed per female per day (Fig. 2C). Additionally, *A. phytoseiuli* caused a significant reduction of fecundity from 18.90 to 2.76 eggs per infected female within the first week (Fig. 2D). However, “nonresponding syndrome” to prey was not found in infected predators (Fig. 2E). Surprisingly, infected males transmitted the pathogen to females during copulation, resulting in female mortality (Fig. S2). The *A. phytoseiuli* infection could be transferred to the next generation through either female or male parents (Fig. S3), while infected females passed a higher dose of *A. phytoseiuli* than infected males to offspring.

### The *A. phytoseiuli* infection targets the reproductive system of *N. californicus* females.

**FIG 3:**
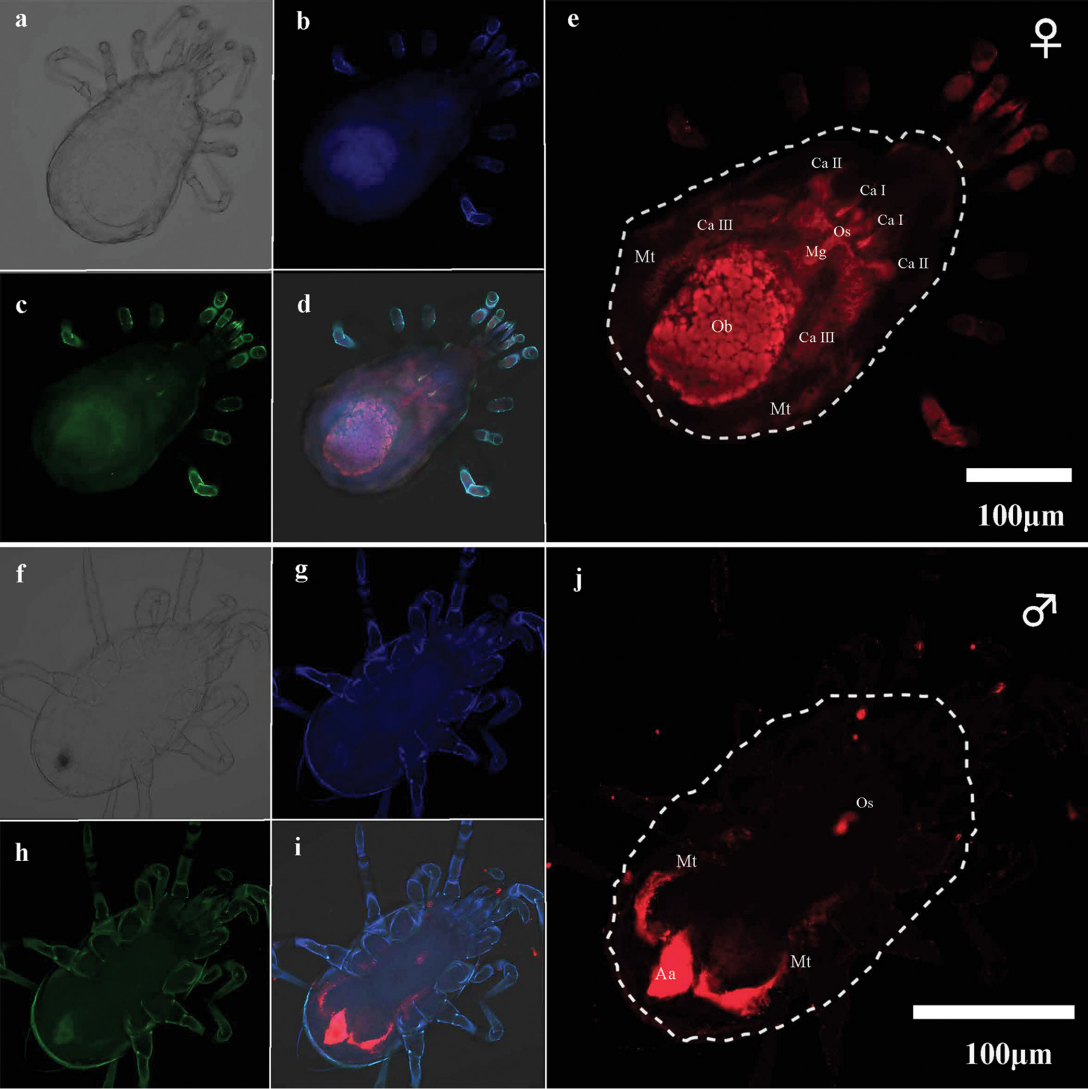
Accumulated locations of *A. phytoseiuli* in two sexes of *N. californicus* by FISH. Adult female (a to e) and male (f to j) mites were observed under ×40 magnification from the dorsal side. Blue, red, and green signals were used for host nuclei (b and g), *A. phytoseiuli* (e and j), and all bacteria (c and h), respectively. Abbreviations: Os, esophagus; Ca I to III, ceca I to III; Mt, Malpighian tubules; Mg, midgut; Ob, oviductal bulb; Aa, anal atrium.

To clarify the enriched location of the pathogen, we performed fluorescence *in situ* hybridization (FISH) of *A. phytoseiuli* in both infected males and females. After 3 days since the mites had been sprayed with *A. phytoseiuli* suspension, the pathogen was widely distributed in the esophagus (Os), three cecal pairings (Ca I to III), central midgut (Mg), Malpighian tubules (Mt), and especially in the oviductal bulb epithelium (Ob) of females (Fig. 3e). In males, *A. phytoseiuli* was confined to the Os, Mt, and anal atrium (Aa) (Fig. 3j).

### The *A. phytoseiuli* infection may transmit through eggshells and to other arthropods.

**FIG 4:**
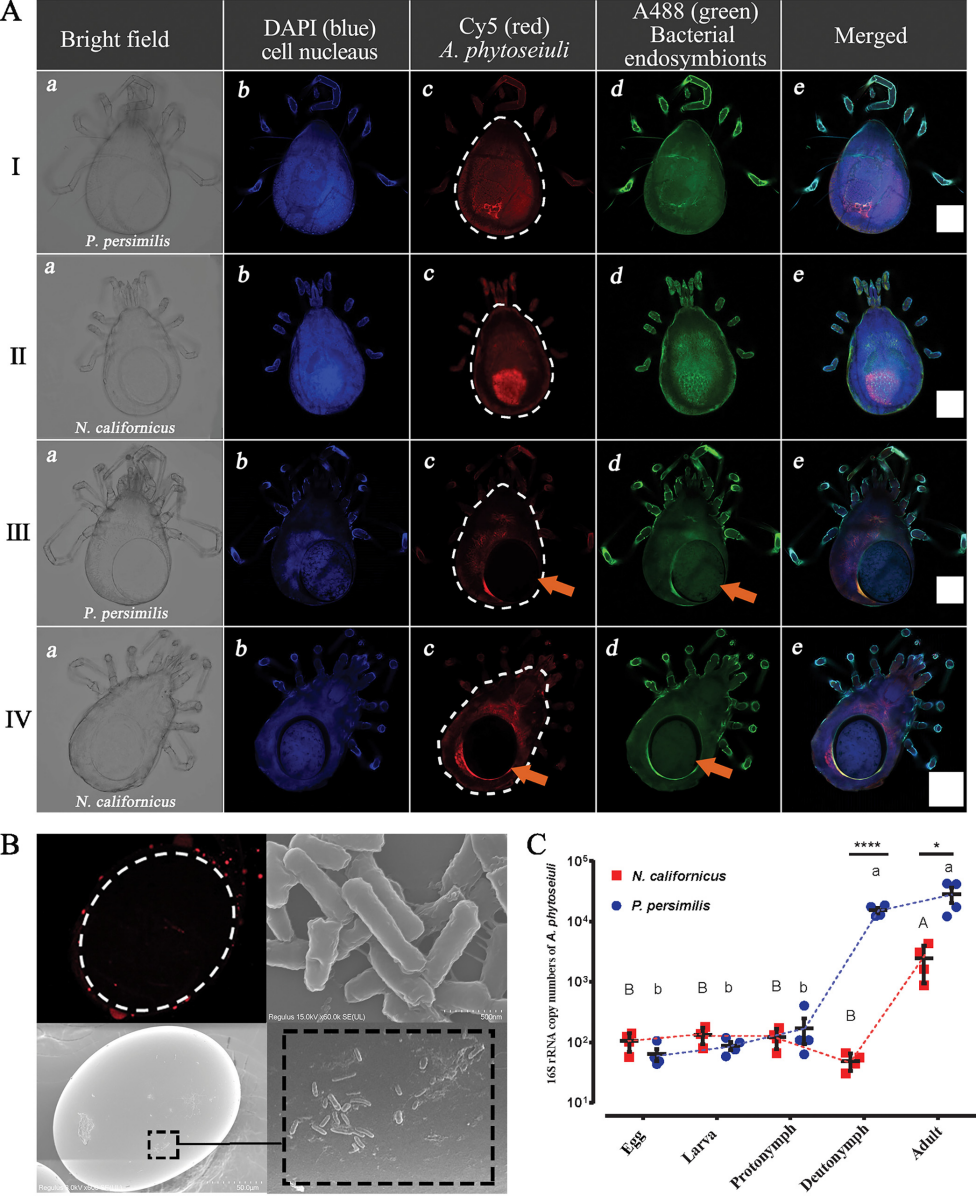
Transgenerational transmission and accumulation of *A. phytoseiuli* in two mite species. (A) FISH images of gravid *P. persimilis* and *N. californicus* from the dorsal side are displayed in 5 different channels: (a) bright field, (b) DAPI (blue) for host nuclei, (c) Cy5 (red) for *A. phytoseiuli*, (d) Alexa Fluor 488 (A488 [green]) for all bacterial endosymbionts, and (e) four merged channels. Rows I and III are *P. persimilis*, and rows II and IV are *N. californicus*. The white dotted outline indicates the mite body shape. The orange arrow indicates the existence of bacteria but not *A. phytoseiuli* in internal eggs. The white scale bars are 100 μm. (B) FISH and SEM images of newly laid eggs of *N. californicus*. Red signals (Cy5 labeled) and short bacilli are both present on eggshell. (C) Copy number of 16S rRNA of *A. phytoseiuli* in five developmental stages of *P. persimilis* and *N. californicus*. Different uppercase and lowercase letters indicate significance among stages in *N. californicus* and *P. persimilis*, respectively. The asterisks indicate comparisons of specific stages between two species. ****, *P* < 0.0001; *, *P* < 0.05.

Given the presence of vertical transmission, we were interested in testing whether *A. phytoseiuli* showed transovarial transmission. FISH images indicate that *A. phytoseiuli* did not exist in ventrally located eggs of both *P. persimilis* and *N. californicus* females, but only on the eggshell (Fig. 4A, panels IIIc and IVc). After eggs had been laid, *A. phytoseiuli* was still on the surface of the egg (Fig. 4B), and then it entered and proliferated in adult predatory mites (Fig. 4C). As mentioned above, *A. phytoseiuli*-free females can become infected by the pathogen when copulating with infected males (Fig. S2). In this case, the pathogen can also pass on the infection to offspring (Fig. S3).

We also examined the potential transfer of *A. phytoseiuli* to other species. We therefore sprayed *A. phytoseiuli* suspension on selected arthropod pests that predatory mites often encountered in the same ecosystem, including two-spotted spider mites (Tetranychus urticae), diamondback moths (Plutella xylostella), whiteflies (Bemisia tabaci), western flower thrips (Frankliniella occidentalis) and bean aphids (Megoura japonica). After spraying 3 days, *A. phytoseiuli* could be detected in *T. urticae* (3/10), *P. xylostella* (2/10), *B. tabaci* (5/10), *F. occidentalis* (6/10), and *M. japonica* (2/10). The proportion of *A. phytoseiuli*-positive adults increased in all species across time (Fig. S4), although disease symptoms were not detected.

## DISCUSSION

In our study, we uncovered the successful spread of the pathogen *A. phytoseiuli* from the commercial predator *P. persimilis* to an indigenous species, *N. californicus.* While *A. phytoseiuli* was thought to be a restricted pathogen only in *P. persimilis* (5), we show here that it may have a potentially wider host range. Given that the genomic identity of the isolate from our study was over 99.8% with the *A. phytoseiuli* infection characterized previously, we consider the strains in China and Europe to be the same. So far, there are no reports of *A. phytoseiuli* infection of *P. persimilis* populations in America and Australia or in other predatory mite species (5), and we also have not detected *A. phytoseiuli* in several wild populations of *N. californicus* in China. We therefore hypothesize that *A. phytoseiuli* may be introduced through *P. persimilis* strains from Europe, with the capacity to be horizontally transferred to *N. californicus* following its close contact with *P. persimilis*.

The transfer of the infection to *N. californicus* was accompanied by severe morphological damage to the mites, involving a shrunken body, reduced prey consumption, early cessation of oviposition, and early death. The disease appeared particularly detrimental to female adults where the pathogen accumulated. In mass-reared *P. persimilis*, *A. phytoseiuli* can induce a “nonresponding syndrome” and reduce predation efficiency (15). However, we found that it did not affect the foraging behavior of *N. californicus* attacking spider mites.

The negative effect of *A. phytoseiuli* on the maternal reproduction system of predatory mites may relate to direct or indirect impacts on mite tissues. Schütte et al. (6) have found that *A. phytoseiuli* accumulated in the lumen of the alimentary tract and may even block it, while the epithelium of the alimentary tract was degenerated. The infection also invaded other tissues, such as the fat body and hemolymph. In our study, we noted effects on the reproductive system of *N. californicus* that included accumulation in the female oviductal bulb. The epithelial cells of the oviduct play an important role in the developing gamete/embryo by secreting various growth factors (16). We assume that *A. phytoseiuli* is entering the alimentary tract by feeding and degrading the epithelium of the lumen before targeting and proliferating in the oviductal bulb.

While tissue destruction associated with the pathogen would prevent vertical transmission intracellularly, the eggshell route of transmission used by the pathogen seems effective, particularly given that transmission can occur via both male and female parental mites. A comparable vertical transmission can be seen in the tortoise leaf beetle, Cassida rubiginosa, where Salem et al. reported that a bacterial symbiont, *Stammera*, only existed in the spheres of the egg caplet of *C. rubiginosa* during the egg stage; thus, the vertical transmission of the symbiont may follow ingestion by an emerging larva (17). However, in the adult stage of *C. rubiginosa*, the *Stammera* infection accumulated in the foregut as well as in the female reproductive tract. During copulation, the spermatophore of the host mite is an isolated structure that is then transferred to the spermatodactyl tip at the bottom of the chelicerae before entering the insemination pore and the spermatheca of the female (18). With the pathogen likely to be present in the spermatophore, this may be the route of pathogen transfer to uninfected females and their reproductive organs.

Through both vertical and horizontal transmission, *A. phytoseiuli* might be expected to be common in predatory mite populations, suggesting a high risk of spread in mass production of predatory mites. Feces and dead mites containing *A. phytoseiuli* can be key sources for infestation of populations (19). In our study, *A. phytoseiuli* could easily enter other insect bodies on leaves with a pathogen suspension. On the one hand, food could be unignorable media to transmit pathogen from prey to predators when feeding. On the other hand, when environmental humidity is suitable, prey fecal bacteria are diluted and active on the leaf surfaces, and from there they could be picked up by other arthropods through natural body openings or the integument (19). According to the “predator prey models with infectious diseases” (20), the predator’s population could acquire an infection from the prey population through the predation process (21). The pathogen may therefore represent a potential source of infection to wild predatory mites if infected *P. persimilis* mites are released for biological control. Interestingly, *A. phytoseiuli* has also been found in three social spiders collected in South Africa, although it does not represent a core component of the microbiota of those species (22). Because the similarity between this *Acaricomes* strain and the DSM14247 *A. phytoseiuli* strain was 98%, pathogenic *A. phytoseiuli* may also to spread to spider host populations.

Invasive mosquitoes, ticks, and mites feeding on host fluids can transmit pathogens to affect mammals and birds, while worldwide invasion by the commercial European honey bee Apis mellifera accelerated the spread of Varroa destructor, the vector of bee viruses and bacteria that kill wild bee colonies (23). We suspect that the current study represents one of the first reports of an introduced invertebrate predator carrying and spreading pathogens in a new region. Once the pathogen infects stocks used in the production of predatory mites, it is possible that the pathogen could spread to natural environments. The pathogen therefore should be routinely monitored during the production of predatory mites. Invasive microbial pathogens might be an increasingly important issue over the next 2 decades (24), and any new introductions of biological control agents should include an evaluation of pathogen risks and their possible ecological impact (25).

## MATERIALS AND METHODS

### Survey of *A. phytoseiuli* in two predatory mite species.

*N. californicus* was collected in Dinghushan Natural Reserve, Guangdong, China, in 2010 (10). *P. persimilis* was shared by a local institute from 2006. These two predatory mite populations were maintained in the Laboratory of Predatory Mites, Institute of Plant Protection, Chinese Academy of Agricultural Sciences, Beijing, China, over a decade. Both species were fed with Tetranychus urticae (Koch) from Phaseolus vulgaris L. Five *P. persimilis* isofemale lines and 10 *N. californicus* isofemale lines used in the present study were separately maintained in plastic boxes, each containing hundreds of individuals (26). All rearing boxes were placed in the incubator under 25 ± 1°C and 70% ± 5% rH with a 16-h/8-h light/dark photoperiod.

The total DNA from 10 adult mites from each line was extracted using a Qiagen DNeasy blood and tissue kit (catalog no. 69506). Predatory mites were surface sterilized with 70% ethanol for 30 s and 0.5% sodium hypochlorite solution for 30 s before being rinsed 3 times in sterile water for another 30 s. Microbial community profiling was performed using the V4 region of the bacterial 16S rRNA gene with primers of 515F and 806R by DNA polymerase (Phusion HF DNA polymerase M0530S; NEB). The purified and barcoded amplicon libraries were pooled on an Illumina NovaSeq 6000 system for PE250 paired-end sequencing (Sinobiocore, Inc., Beijing, China). After filtering of low-quality reads, forward and reverse reads were joined to assign zero-radius operational taxonomic units (zOTUs) using a 97% similarity cutoff in USEARCH UPARSE (27). All zOTUs presented in less than 0.1% abundance were removed. The samples were rarefied at a depth of 10,000 reads per sample. The relative abundance of *A. phytoseiuli* in 5 *P. persimilis* lines and 10 *N. californicus* lines was determined from the percentage of *A. phytoseiuli* reads among the rarefied 10,000 total reads per sample.

### Isolation and genome sequencing of *A. phytoseiuli* from *N. californicus*.

Based on the microbiome results, we selected isofemale lines for sequencing from both *P. persimilis* and *N. californicus* that were infected by *A. phytoseiuli* and homogenized 10 surface-sterilized female adults. We then diluted homogenates to 200 μL and plated them onto tryptic soy agar (TSA) plates before incubating them at 25°C for 14 days. The *A. phytoseiuli* colonies had a smooth, circular and yellowish morphology 1 to 2 mm in diameter at 12 days (14). PCR amplicons of the isolated colonies were amplified using universal primers 27F and 1492R of the 16S rRNA gene (see Table S4 in the supplemental material) before Sanger sequencing (Sangon Biotech, Shanghai, China). *A. phytoseiuli*-specific primers Ap1-F and Ap1-R were designed for further microbial identification (Table S4).

Genomic DNA of the *A. phytoseiuli* ZQ strain was extracted from a colony isolated from *N. californicus* by a Qiagen DNeasy kit and then submitted to the Illumina NovaSeq 6000 system for PE250 paired-end sequencing (Sinobiocore, Inc., Beijing, China), resulting in 4,665,212 raw reads and finally 99.51% quality control (QC) reads. Reads were assembled using Edena v.3.131028 (28) with default parameters, producing 146 contigs and a 2.37-Mb genome size. A total of 2,194 genes were predicted by Prodigal v.2.6 (29). Virulence factors were searched using BLAST against the VFDB (30) and PHI-base (31).

### Phylogenetic analysis.

Besides one sequence from our study, a total of 80 sequences of 16S rRNA from the family *Micrococcaceae* and two sequences from *Streptomycetaceae* as outgroups were downloaded from NCBI. These sequences were aligned using MUSCLE (32). Poorly aligned positions and divergent regions were eliminated by the Gblocks server (33) with settings of allowed gap positions, smaller final blocks, and less strict flanking positions. The final alignment was used to build a maximum likelihood phylogeny using IQ-TREE with an automatically determined best-fit model and 1,000 bootstrap replicates (34).

### Transfer of *A. phytoseiuli* from *P. persimilis* to *N. californicus*.

Based on microbiome profiling and PCR identification of *A. phytoseiuli*, we identified five infected *P. persimilis* lines and five uninfected *N. californicus* lines to test pathogen transfer. We combined two randomly selected lines (one line from each species) in another new rearing box used for normal mite rearing. This produced five combinations of lines that were considered five biological replicates. Controls consisted of five lines of each species kept separately. After 2 weeks, 10 adults from each box were sampled to characterize *A. phytoseiuli* prevalence by extracting DNA and PCR amplification as described above.

### Bioassays on pathogenicity of *A. phytoseiuli* isolated from *N. californicus*.

One *A. phytoseiuli* colony was transferred from TSA plates to TSB in an incubator shaker at 25°C for 180 rpm. After 2 to 3 days, the *A. phytoseiuli* culture was collected, and the cells were dispersed in 1× phosphate-buffered saline (PBS) buffer. The optical density at 600 nm (OD_600_) was adjusted to 1, corresponding to *A. phytoseiuli* at 1.76 × 10^8^ CFU/mL.

### (i) Survival assays.

A solution of *A. phytoseiuli* cells was prepared to spray mites. Half of the solution was heated at 80°C for 30 min and considered a heat-killed bacterial suspension; plating on TSA confirmed the absence of living cells. Sixty *N. californicus* predatory mites for each of the three treatments (live and heat-killed *A. phytoseiuli* suspensions as well as PBS) and 40 *N. californicus* mites assigned as the control (no spray) were used to assess pathogen impact on survival rate. Mites were reared from egg stage in a rearing chamber, which was described by Zhang et al. (35). The bacterial suspension or PBS was sprayed evenly over the surface of 4-cm-diameter bean leaf discs by a handheld sprayer every day. The number of live mites was recorded daily, and dead bodies were collected and stored in ethanol immediately for further confirmation of the presence of the pathogen. Survival curves and statistical analysis using a pairwise log rank test was completed in Prism (GraphPad).

### (ii) Predatory capacity of *N. californicus* infected with *A. phytoseiuli*.

More than 100 female deutonymph mites from an uninfected population of *N. californicus* were randomly divided into two groups and sprayed with the *A. phytoseiuli* suspension (OD_600_ = 1) or PBS for 3 days. The individuals used for prey consumption were picked from these two groups. To detect the predatory efficiency of adult mites, we individually transferred young female adults starved for 24 h to rearing chambers containing 10 spider mite eggs. We then recorded the remaining eggs number after 24 h. A new chamber with 10 eggs was provided for each predatory mite every day for successive 7 days.

### (iii) Fecundity of *N. californicus* infected with *A. phytoseiuli*.

To test whether males or females were more affected by the infection, the treatments of *N. californicus* deutonymph mites of both sexes were repeated, but mites were mated after emergence to adults in four combinations: *A. phytoseiuli-*free female × *A. phytoseiuli-*free male (♀− × ♂−), *A. phytoseiuli-*free female × *A. phytoseiuli-*infected male (♀− × ♂+), *A. phytoseiuli-*infected female × *A. phytoseiuli*-free male (♀+ × ♂−), and *A. phytoseiuli-*infected female × *A. phytoseiuli-*infected male (♀+ × ♂+). Males were removed after 1 day to ensure that females could be observed better. Daily fecundity of each female on a leaf disc was recorded, while leaf discs were changed daily to ensure sufficient prey.

### (iv) Predatory behavior of *N. californicus* infected with *A. phytoseiuli*.

To examine whether the pathogen affected predatory behavior, 20 young females of *N. californicus* as a group were used to develop a predatory index (−1 to 1). The predatory index of each group was calculated by the formula (no. of mites selected *T. urticae* – no. of mites selected clean leaf)/20. A *T. urticae-*infected leaf disc and a clean leaf disc separated by 10 cm were placed in a clean petri dish, and the group of 24-h-starved *N. californicus* mites was then introduced between the two leaf discs. If all predatory mites in the group chose the *T. urticae-*infected leaf disc, it was scored as 1, and if all predatory mites chose the clean leaf disc, it was scored as −1. Eleven and 14 replicate groups were tested for *A. phytoseiuli-*infected mites (treatment) and *A. phytoseiuli-*free mites (PBS), respectively. Because data were normally distributed, an independent *t* test was used to compare treatments using Prism (GraphPad).

### FISH to detect *A. phytoseiuli* in *P. persimilis* and *N. californicus* adults.

Whole-mount FISH analyses followed published protocols (36). Briefly, newly mated adults were sampled and fixed in Carnoy’s solution overnight before undergoing bleaching in 6% hydrogen peroxide in 80% ethanol to quench autofluorescence. After being hybridized overnight, samples were mounted in SlowFade Diamond antifade mountant with DAPI (4′,6-diamidino-2-phenylindole) (Thermo Fisher Scientific) and observed under a confocal laser-scanning microscope (Zeiss 980). The information on fluorescent dyes and wavelengths is provided in Table S5. We inspected 10 adult predatory mites per species or per sex and picked a single representative mite to perform a z-stack capture using constant laser intensity and aperture. Images were acquired using Zeiss ZEN microscope software.

### Scanning electron microscopy of *A. phytoseiuli* and *N. californicus* eggs.

We collected *A. phytoseiuli* cells by rinsing with 1× PBS (pH 7.0) and removing the supernatant. We also sampled newly laid eggs produced by infected *N. californicus* females. Samples were fixed overnight by 2.5% glutaraldehyde solution at 4°C and rinsed with 1× PBS three times. We then added 500 μL of 1% osmic acid to fix for an hour and dehydrated by ethanol solution with a gradient concentration from 20 to 50 to 80 to 100%. Pure acetone was finally used to replace 100% ethanol, and the samples were air dried. Over 20 samples of *A. phytoseiuli* and *N. californicus* eggs were then coated with platinum (Leica EM ACE600) and observed under 3 kV (Hitachi Regulus 8100).

### Real-time qPCR assay on *P. persimilis* and *N. californicus*.

Real-time quantitative PCR (qPCR) was performed to detect titers of *A. phytoseiuli* at different developmental stages of infected *P. persimilis* and *N. californicus* isofemale lines. Thirty eggs or nymphs and 10 adults were collected from each line and surface sterilized for DNA extraction. The concentrations of all DNA samples were measured by a Qubit 4 fluorometer (Thermo Fisher Scientific) before dilution to the same concentration. Four biological repeats and three technical replicates were undertaken for each sample. Copy numbers of 16S rRNA were determined by standard curves covering the range from 10^2^ to 10^7^ copies with *R*^2^ above 0.99. Real-time qPCR assays were run in an Applied Biosystems QuantStudio 5 (Thermo Fisher Scientific).

### Data availability.

The entire genome of *A. phytoseiuli* has been deposited at GenBank under BioProject no. PRJNA886762. Two 16S rRNA sequences of *A. phytoseiuli* were uploaded in GenBank under accession no. MZ361977 and MZ363838.
