# Supplementary material for: Detection, Detrimental Effects, and Transmission Pathways of the Pathogenic Bacterium Acaricomes phytoseiuli in Commercial Predatory Mites
Source: Microbiol Spectr. 2022 Nov 2;10(6):e02654-22. doi: 10.1128/spectrum.02654-22 (PMC9769712; doi:10.1128/spectrum.02654-22)
Supplement: Supplemental file 1 — Fig. S1 to S4 and Tables S1 and S3 to S5. Download spectrum.02654-22-s0001.pdf, PDF file, 0.5 MB [file spectrum.02654-22-s0001.pdf]

Supplementary Information

Detection, detrimental effects and transmission pathways of the pathogenic bacterium *Acaricomes phytoseiuli* in commercial predatory mites

Zhongqiu Xie, Ary A. Hoffmann, Bo Zhang, Xuenong Xu

Fig. S1. Complete ML tree of the family of Micrococcaseae based on 16S rRNA sequences. The red branches indicate *A. phytoseiuli*.

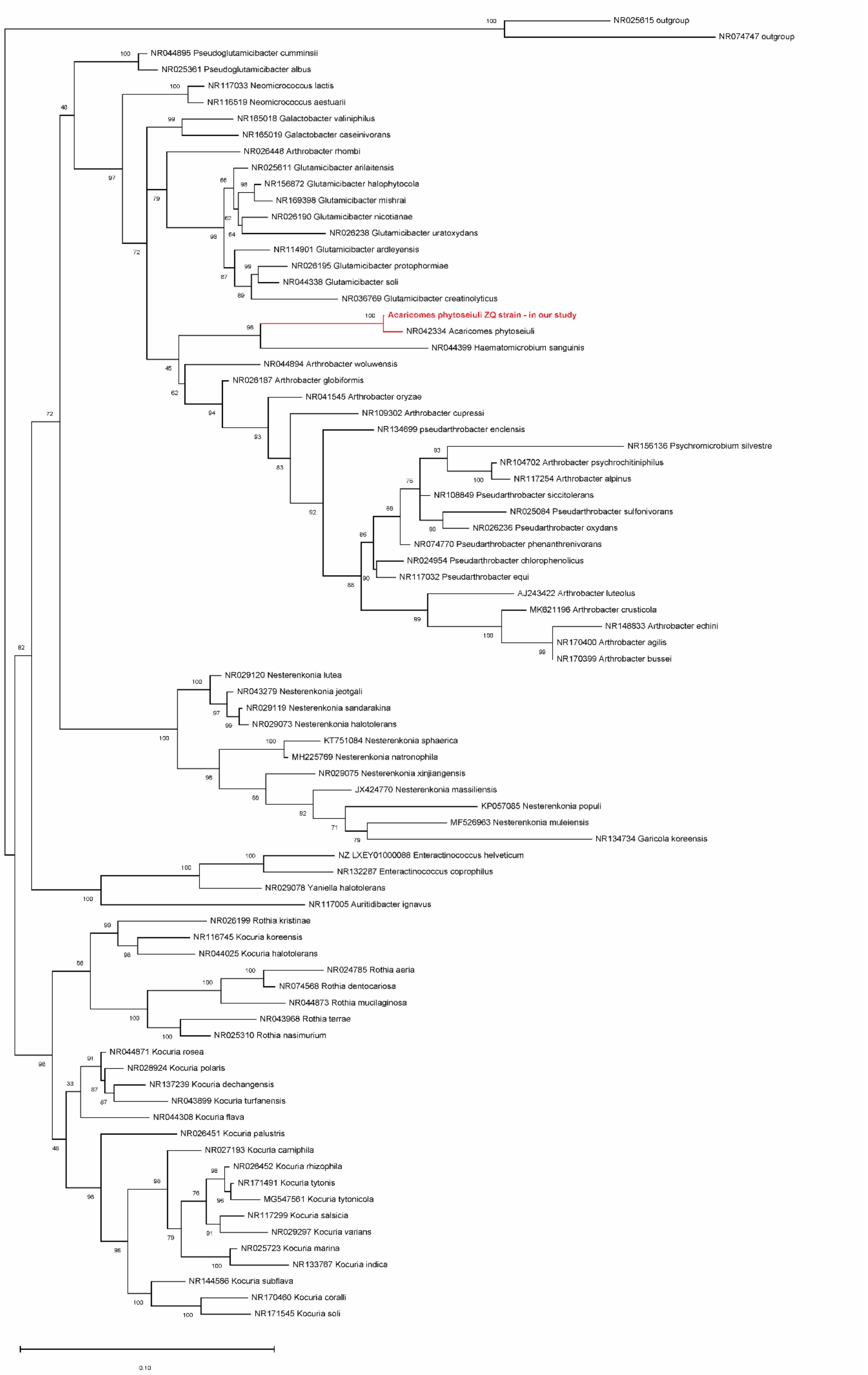

Fig. S2. Survival comparison of females of *N. californicus* after copulating with males with/without the *A. phytoseiuli* infection. The p-values were obtained using a log-rank test with Mantel-Cox test correction. ****=P < 0.001, ** = p < 0.01, NS= not significant.

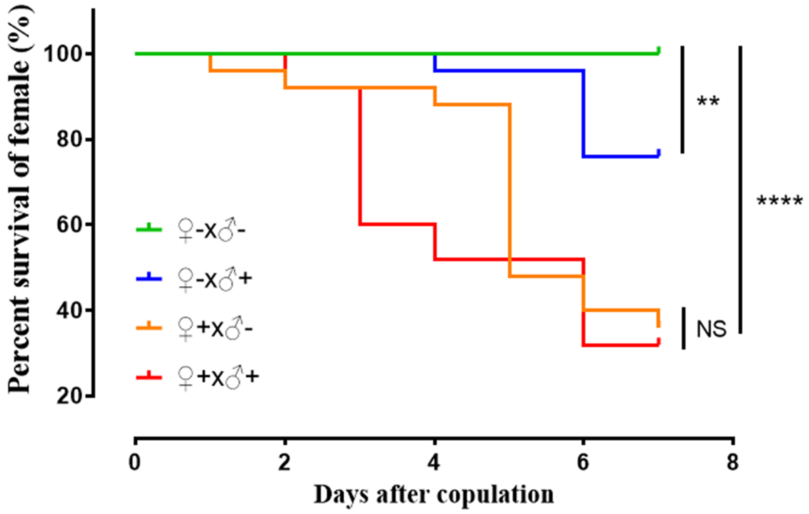

Fig. S3. The relative fold changes of *A. phytoseiuli* in the offspring produced by pathogen infected parents of *N. californicus*. Treatments were compared with independent t-tests after log2 transformed data. *** indicates $p < 0.001$.

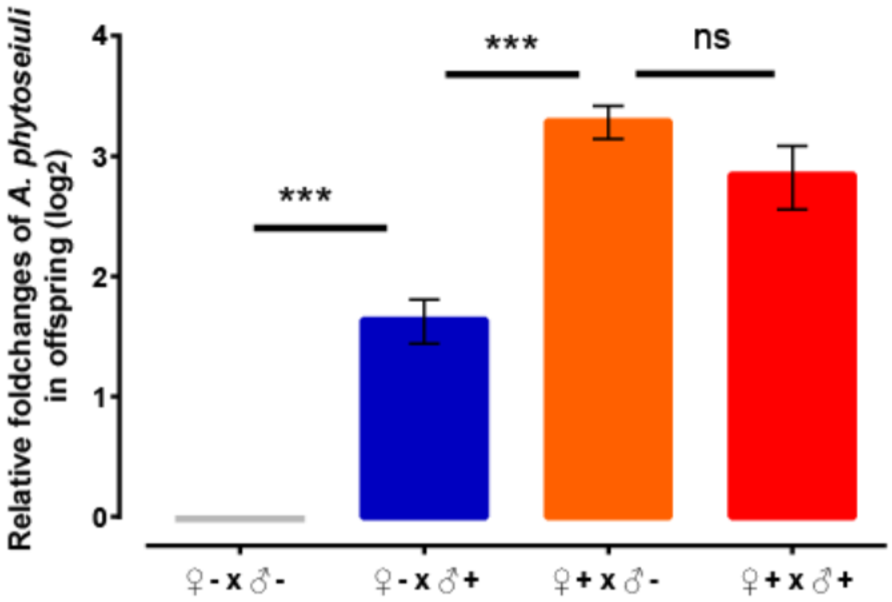

Fig. S4. The percentage of insects (*Megoura japonica*, *Bemisia tabaci*, *Frankliniella occidentalis* and *Plutella xylostella*) as well as a mite (*Tetranychus urticae*) that became infected with *A. phytoseiuli* after exposure to the pathogen in a suspension.

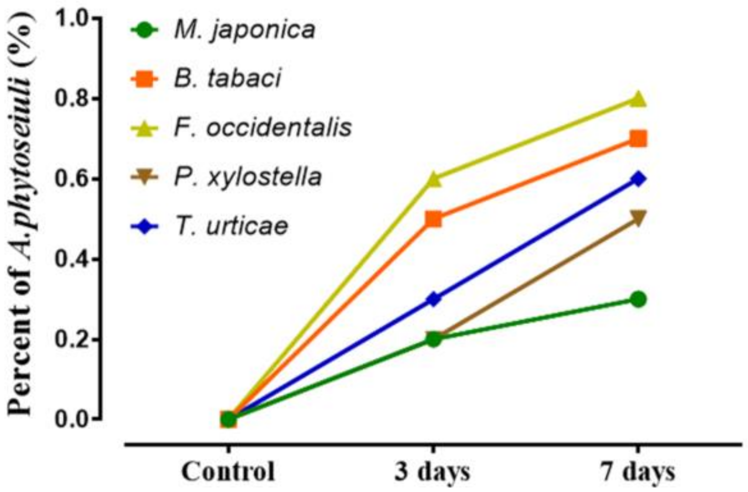

Table S1. Genome assembly and quality data for *A. phytoseiuli* ZQ strain in our study and DSM14247 from NCBI.

<table>
<tr><th></th><th>Contigs</th><th>N50</th><th>Avg contig length</th><th>Max contig length</th><th>Genome size</th><th>%GC</th><th>CDS</th><th>rRNA</th><th>tRNA</th><th>Other RNA</th><th>Average nucleotide identity</th></tr>
<tr><td>ZQ strain</td><td>146</td><td>43,726</td><td>16,203</td><td>101,176</td><td>2.37 Mb</td><td>62.47</td><td>2194</td><td>5</td><td>48</td><td>1</td><td rowspan="2">99.78%</td></tr>
<tr><td>DSM14247</td><td>64</td><td>91,146</td><td>--</td><td>--</td><td>2.42Mb</td><td>62.3</td><td>2138</td><td>6</td><td>45</td><td>3</td></tr>
</table>

Table S2. Virulence factors predicted by VFDB and PHI-base.
The table is an excel file.

Table S3. Sampling information of local Chinese *N. californicus* populations.

**Origin location**	**Host plant**	**Date and collector**	**Coordinates**	**#Samples**
Yongshan County,<br>Yunnan Province	*Citrus reticulata* Blanco	April 2021<br>Xuenong Xu	E 104.07762<br>N 30.09019	45
Zhaoyang District,<br>Yunnan Province	*Malus pumila*	November 2020<br>Wei Zhang	E 103.65234<br>N 27.21750	89
Dinghushan Natural Park,<br>Guangdong Province	*Yulania denudata*	April 2021<br>Xuenong Xu	E 112.54537<br>N 23.17031	96
Fuzhou City,<br>Fujian Province	--	--	E 119.27345<br>N 26.04769	15

Table S4. Primers and annealing temperatures used for *A. phytoseiuli* detection.

**Primer name**	**Sequence**	**Length (bp)**	**Tm (°C)**
V4 region	515F: GTGCCAGCMGCCGCGGTAA<br>806R: GGACTACNNGGGTATCTAAT	291	55
Full 16S sequences	27F: AGAGTTTGATCCTGGCTCAG<br>1492R: TACGGCTACCTTGTTACGACTT	1465	55
Specified primers of *A. phytoseiuli*	Ap1-F: ACGTGAGTAACCTGCCCAAG<br>Ap1-R: CCACCGCTACACCAGGAATT	564	57
qPCR primers of *A. phytoseiuli*	F: ACGTGAGTAACCTGCCCAAG<br>R: GGCCATTACCCCACCATCAA	148	60
FISH probe of *A. phytoseiuli*	GCGATCTAGGCGATGTCAAGCC<br>5`CY5		
FISH probe Eub339	TGCTGCCTCCCGTAGGAG<br>5`ALEXA FLUOR 488		

Table S5. Wavelengths of three fluorescent dyes used for *A. phytoseiuli* detection.

**Dye Name**	**Fluorescent color**	**Laser line**	**Excitation max**	**Emission max**
DAPI	Blue	405 nm	350 nm	470 nm
Cy5	Red	633 nm/ 647 nm	649 nm	666 nm
A448	Green	488 nm	490 nm	525 nm
